# *Takakia* possesses a key marker of embryophyte sporopollenin

**DOI:** 10.17912/micropub.biology.001165

**Published:** 2024-07-26

**Authors:** Dae-Yeon Suh, Damanpreet K Sraan, Neil W Ashton

**Affiliations:** 1 Chemistry and Biochemistry, University of Regina, Regina, Saskatchewan, Canada; 2 Biology, University of Regina, Regina, Saskatchewan, Canada

## Abstract

The enigmatic moss, *Takakia lepidozioides* , possesses a particular type III polyketide synthase, ASCL (Anther-Specific Chalcone synthase-Like), that is an identifying marker for genuine sporopollenin in the walls of embryophyte spores and pollen grains. By contrast, a survey of all algae with sequenced genomes confirms that they do not possess ASCL and, therefore, their spore walls are not composed of sporopollenin.

**Figure 1. Maximum Likelihood tree of plant type III polyketide synthases:**
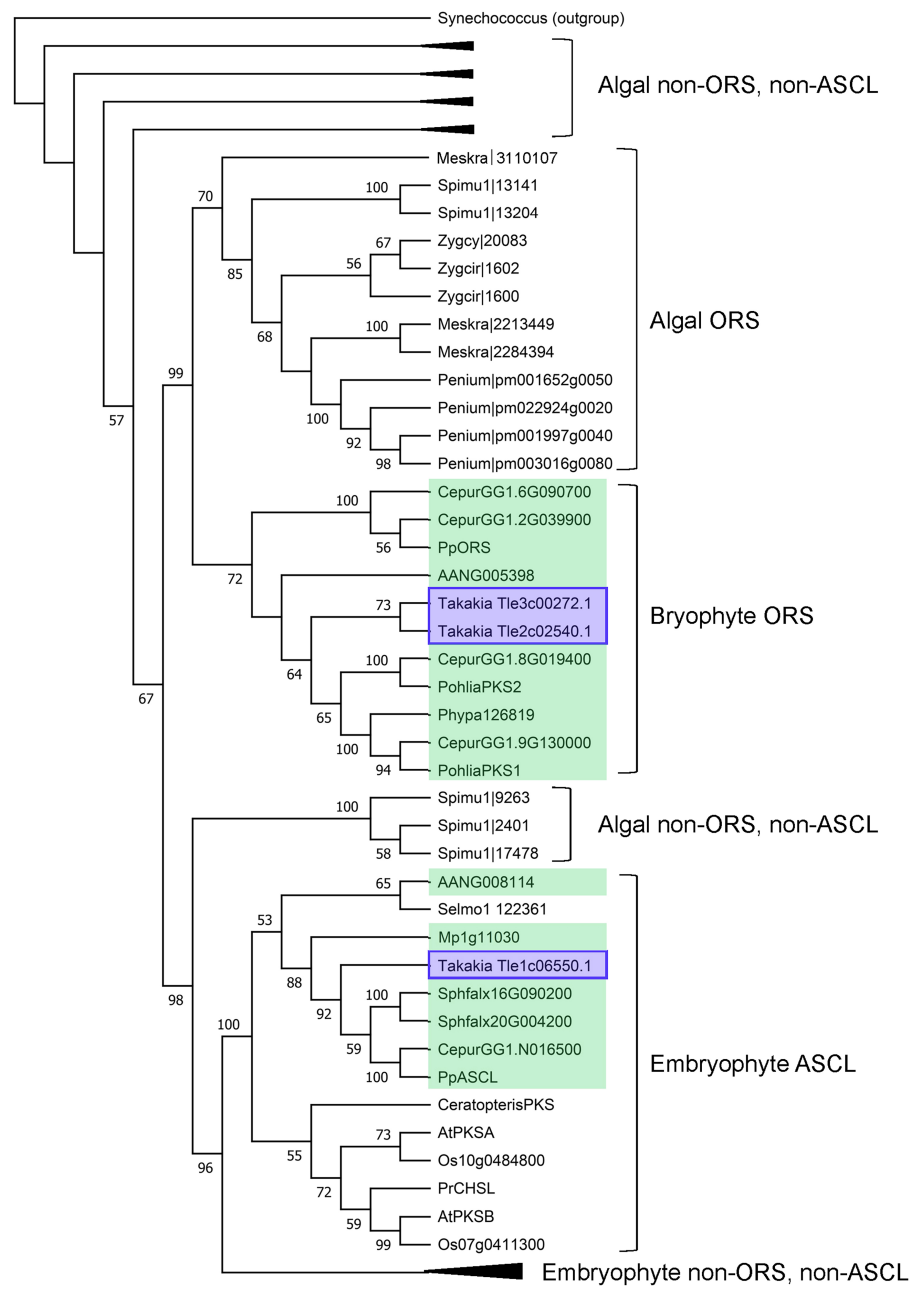
Included in the tree are sequences of all known algal type III polyketide synthases, all known bryophyte enzymes that belong to either ORS (2ꞌ-OxoalkylResorcinol Synthase) or ASCL clades, representative ASCL enzymes from other major embryophyte groups, and some non-ORS and non-ASCL type III PKS enzymes from diverse embryophyte taxa. *Takakia* enzymes and other bryophyte enzymes are highlighted in blue and green, respectively. Bootstrap values (>50%) are displayed at the nodes. A cyanobacterial type III PKS was used as outgroup to root the tree. For brevity, four of the five algal non-ORS and non-ASCL clades and the embryophyte non-ORS and non-ASCL clade, which includes >20 diverse enzymes, e.g. chalcone synthase, stilbene synthase (Shimizu et al. 2017), have been collapsed. Sequences used for tree construction are provided in the Extended Data section (Fig. S1).

## Description

Sporopollenin (SP) is the chemically resistant wall material of plant spores and pollen, which provides protection from subaerial stresses. For most of the time since the term was coined by Zetzsche in 1931 (Zetzsche and Kälin, 1931; Zetzsche and Vicari, 1931) resistance to acetolysis has been the sole criterion for the identification of SP. This chemically imprecise definition has resulted in numerous claims for the existence of SP or SP-like material in algae and various microorganisms (Suh and Ashton 2022 and references therein). Based on more recent chemical analyses (reviewed by Grienenberger and Quilichini 2021), the current view of embryophyte SP is that it is a polymer composed of polyhydroxylated polyketides, hydroxylated aromatics and fatty acid derivatives, crosslinked via ester and ether bonds and oxidative C–C coupling. A particular type III polyketide synthase (PKS), ASCL, plays a central role in the polyketide biosynthetic pathway that provides hydroxylated polyketides as SP precursors (Kim et al. 2010; Colpitts et al. 2011; Suh and Ashton 2022) . Following an extensive BLASTp survey of plant genomes, we discovered that with an exception, the marine monocot, *Zostera marina* , which has exineless pollen (Olsen et al. 2016) , representatives of all major embryophyte clades possess ASCL in stark contrast to algae, including Charophytes, which do not. This led us to propose a new definition for SP as follows to distinguish genuine SP in embryophytes from SP-like compounds such as the acetolysis-resistant algaenans possessed by some Chlorophytes and to use ASCL as an identifying marker for the presence of genuine SP in spore and pollen grain walls (Suh and Ashton 2022) .

‘Sporopollenin is a chemically resistant complex heteropolymer present in the outer walls of spores and pollen grains and is composed partly of hydroxylated polyketides derived from the conserved polyketide pathway, which involves ASCL.’

At the time we made this definition, the genome of the phylogenetically enigmatic plant, *Takakia* , of which there are only two species, *Takakia lepidozioides* and *Takakia ceratophylla* , was not available. Relatively recently, *Takakia* has been shown to be sister to all other extant mosses (Liu et al. 2019) and to have diverged from the Last Common Ancestor of embryophytes after hornworts and liverworts (Hu et al. 2023) . Therefore, we were keen to discover whether the recently sequenced *Takakia lepidozioides* genome possesses an *ASCL* gene like the vast majority of other embryophyte genomes, indicating the probable presence of genuine SP in its spore wall.

Putative ASCL and other type III PKS sequences in *Takakia* were identified based on phylogeny and sequence analysis. Our Maximum Likelihood (ML) phylogenetic tree ( Fig. 1 ) resolves type III PKS sequences into the following clades: **(a)** an embryophyte ASCL clade containing bryophyte ASCL sequences including one *Takakia* sequence plus ASCL sequences from other major embryophyte groups. In agreement with the other ASCL sequences, the *Takakia* ASCL possesses diagnostic sequence features in addition to those for type III PKSs, namely Gly225 and (Ala/Val)240 (numbering based on PpASCL (Colpitts et al. 2011) ) (Fig. S1), **(b)** a bryophyte clade containing exclusively ORS sequences including two *Takakia* sequences. In agreement with the other ORS sequences, the *Takakia* ORSs possess diagnostic sequence features in addition to those for type III PKSs, namely Gln218, (Val/Ala)277 and Ala286 (numbering according to PpORS (Kim et al. 2013) ) (Fig. S1), **(c)** an algal clade containing exclusively Charophyte ORS sequences, **(d)** an embryophyte clade comprised of non-ORS and non-ASCL type III PKS sequences including a representative *Takakia* type III PKS, **(e)** five separate algal clades, collectively comprising 35 non-ORS and non-ASCL type III PKS sequences.

Thus, *Takakia lepidozioides* has an *ASCL* gene in agreement with our contention that all embryophytes, with the possible exception of a few species, possess ASCL, which serves as a marker for genuine SP in their spore or pollen walls. The few species predicted to lack ASCL are likely to exist in habitats that do not require protection from subaerial stresses, e.g. *Zostera marina* , and are presumed to have lost ASCL and pollen wall SP secondarily by reductive evolution.

In this study, we have examined all the algal genomes in the PhycoCosm database and reinforced our discovery that, while algae possess type III PKS sequences, none of them falls within the ASCL clade and, therefore, algae don’t possess genuine SP.

Another relevant discovery is that *Takakia* has two full-length *ORS* genes, whose distribution in the Plant Kingdom, unlike that of *ASCL* genes, is limited to Charophytes and bryophytes. Notably, however, *Marchantia* species lack *ORS* genes. As more data become available, it will be interesting to see whether ORS is missing in all liverworts or if the *Marchantia* genus is the lone exception. We have shown previously that ORS is required for integrity of the leaf cuticle of *Physcomitrium patens* and for its resistance to dehydration (Li et al. 2018) and that (2ꞌ-oxo)alkylresorcinols restore dehydration tolerance in a PpORS knockout line (Aslam et al. 2022) . We presume ORS has the same role in *Takakia* and at least some other bryophytes. Interestingly, although ORS sequences are present in Charophytes, they appear to be absent from other algae.

## Methods

We performed BLASTp searches against the *Takakia lepidozioides* genome database ( https://www.takakia.com/blast/blast_cs.html (v3.1)) with PpASCL and PpORS ( *Physcomitrium patens* 2ꞌ-OxoalkylResorcinol Synthase), a bryophyte/charophyte-specific type III PKS, as query sequences. Sixteen putative type III PKS models were identified based on the presence of the catalytic Cys-His-Asn triad and signature sequences (G/A)FGPG (Suh et al. 2000) . Among these sixteen sequences, one ASCL and two ORS sequences were recognised based on phylogeny ( Fig. 1 ) and possession of additional enzyme-specific residues as described in the text and in Fig. S1. Similarly, putative algal type III PKS sequences were retrieved by BLASTp searches against each algal genome in PhycoCosm ( https://phycocosm.jgi.doe.gov/phycocosm/home (accessed on 01 September 2023)). In cases of fusion proteins, portions of sequences that matched type III PKS sequences from the same or related species were taken for further analysis. Representative embryophyte type III PKS sequences were retrieved from Phytozome 13 ( https://phytozome-next.jgi.doe.gov/ ( *Physcomitrium patens* v3.3)) as described previously (Aslam et al. 2022) . The sequences used for tree reconstruction (Table 1) were aligned by the MUSCLE method in MEGA 11 (Tamura et al 2021) , and a ML phylogenetic tree ( Fig. 1 ) was reconstructed in MEGA 11 using the JTT substitution model. The initial tree was created using the default NJ/BioNJ method, and tree improvement was performed using the nearest-neighbor-interchange ML heuristic method. Support for the tree was measured using 1,000 bootstrap replicates.

## Reagents

**Table 1.** Plant type III polyketide synthases used for tree reconstruction

Type III PKSs are listed in the same order of their appearance (from the top) in the ML tree ( Fig. 1 ) before collapsing some of the clades. *Takakia* enzymes are shown in bold. Fusion proteins containing a type III PKS domain are indicated with asterisks.

**Table:** 

Enzyme	Species	Classification
** Outgroup**		
Synechococcus PKS	* Synechococcus* sp.	Cyanobacteria, Synechococcales
** Algal type III PKS (non-ORS, non-ASCL)**		
Chrveli1|20057*	*Chromera velia*	SAR, Chromerida
Pico_ML_1|52161	*Picocystis sp.*	Chlorophyta, Picocystales
Semro1|36990*	*Seminavis robusta*	Ochrophyta, Naviculales
Ochro1393_1_4|754228*	*Ochromonas sp.*	Ochrophyta, Ochromonadales
Ochro2298_1|456847	*Ochromonadaceae sp.*	Ochrophyta, Ochromonadales
Ochro2298_1|419265*	*Ochromonadaceae sp.*	Ochrophyta, Ochromonadales
Ochro1393_1_4|932390	*Ochromonas sp.*	Ochrophyta, Ochromonadales
Ochro1393_1_4|179269	*Ochromonas sp.*	Ochrophyta, Ochromonadales
Mesen1|9713	*Mesotaenium endlicherianum*	Charophyta, Zygnematales
Ectsil1|17490	*Ectocarpus siliculosus*	SAR, Ectocarpales
Claok1|5931	*Cladosiphon okamuranus*	SAR, Ectocarpales
Sacja1|7224	*Saccharina japonica*	SAR, Laminariales
Macpyr2|5041534	*Macrocystis pyrifera*	SAR, Laminariales
Undpi1|10741	*Undaria pinnatifida*	SAR, Laminariales
Alaesc1|15363	*Alaria esculenta*	SAR, Laminariales
MonC141_1|1230	*Monodopsis strain*	Ochrophyta, Eustigmatales
VisC74_1|13231*	*Vischeria strain*	Ochrophyta, Eustigmatales
Ochro1393_1_4|905391	*Ochromonas sp.*	Ochrophyta, Ochromonadales
Ochro2298_1|408009	*Ochromonadaceae sp.*	Ochrophyta, Ochromonadales
Ochro2298_1|458546	*Ochromonadaceae sp.*	Ochrophyta, Ochromonadales
Pelago2097_1|478370	*Pelagophyceae sp.*	SAR, Pelagomonadales
Ectsil1|30595	*Ectocarpus siliculosus*	SAR, Ectocarpales
Claok1|7458*	*Cladosiphon okamuranus*	SAR, Ectocarpales
Macpyr2|9688433	*Macrocystis pyrifera*	SAR, Laminariales
Sacja1|14235	*Saccharina japonica*	SAR, Laminariales
Alaesc1|3438	*Alaria esculenta*	SAR, Laminariales
Undpi1|3722	*Undaria pinnatifida*	SAR, Laminariales
Undpi1|3721	*Undaria pinnatifida*	SAR, Laminariales
SymretSc1|46235	*Symbiochloris reticulata*	Chlorophyta, Trebouxiales
Coccomyxa PKS	*Coccomyxa subellipsoidea*	Chlorophyta, Trebouxiales
Sceob152z_1|1656	*Scenedesmus obliquus*	Chlorophyta, Sphaeropleales
Tetrob172_l|3940502	*Tetradesmus obliquus*	Chlorophyta, Sphaeropleales
Spimu1|9263*	*Spirogloea muscicola*	Charophyta, Spirogloeales
Spimu1|2401*	*Spirogloea muscicola*	Charophyta, Spirogloeales
Spimu1|17478*	*Spirogloea muscicola*	Charophyta, Spirogloeales
** Algal ORS**		
Meskra|3110107	*Mesotaenium kramstae*	Charophyta, Zygnematales
Spimu1|13141	*Spirogloea muscicola*	Charophyta, Spirogloeales
Spimu1|13204	*Spirogloea muscicola*	Charophyta, Spirogloeales
Zygcyl6981a_1|20083	*Zygnema cf. cylindricum*	Charophyta, Zygnematales
Zygcir1559_1|1602	*Zygnema circumcarinatum*	Charophyta, Zygnematales
Zygcir1559_1|1600	*Zygnema circumcarinatum*	Charophyta, Zygnematales
Meskra|2213449	*Mesotaenium kramstae*	Charophyta, Zygnematales
Meskra|2284394	*Mesotaenium kramstae*	Charophyta, Zygnematales
Penium|pm001652g0050	*Penium margaritaceum*	Charophyta, Desmidiales
Penium|pm022924g0020	*Penium margaritaceum*	Charophyta, Desmidiales
Penium|pm001997g0040	*Penium margaritaceum*	Charophyta, Desmidiales
Penium|pm003016g0080	*Penium margaritaceum*	Charophyta, Desmidiales
** Bryophyte ORS**		
CepurGG1.6G090700	*Ceratodon purpureus*	Bryophyta, Dicranales
CepurGG1.2G039900	*Ceratodon purpureus*	Bryophyta, Dicranales
PpORS	*Physcomitrium patens*	Bryophyta, Funariales
AANG005398	*Anthoceros angustus*	Anthocerotophyta, Anthocerotales
**Takakia Tle2c02540.1**	***Takakia lepidozioides***	**Bryophyta, Takakiales**
**Takakia Tle3c00272.1**	***Takakia lepidozioides***	**Bryophyta, Takakiales**
CepurGG1.8G019400	*Ceratodon purpureus*	Bryophyta, Dicranales
Pohlia PKS2	*Pohlia nutans*	Bryophyta, Bryales
Phypa 126819	*Physcomitrium patens*	Bryophyta, Funariales
CepurGG1.9G130000	*Ceratodon purpureus*	Bryophyta, Dicranales
Pohlia PKS1	*Pohlia nutans*	Bryophyta, Bryales
** Embryophyte ASCL**		
AANG008114	*Anthoceros angustus*	Anthocerotophyta, Anthocerotales
Mp1g11030	*Marchantia polymorpha*	Marchantiophyta, Marchantiales
**Takakia Tle1c06550.1**	***Takakia lepidozioides***	**Bryophyta, Takakiales**
Sphfalx16G090200	*Sphagnum fallax*	Bryophyta, Sphagnales
Sphfalx20G004200	*Sphagnum fallax*	Bryophyta, Sphagnales
CepurGG1.N016500	*Ceratodon purpureus*	Bryophyta, Dicranales
PpASCL	*Physcomitrium patens*	Bryophyta, Funariales
Selmo1:122361	*Selaginella moellendorffii*	Lycopodiophyta, Selaginellales
Ceratopteris PKS	*Ceratopteris richardii*	Polypodiopsida, Polypodiales
PrCHSL	*Pinus radiata*	Gymnosperms, Pinales
AtPKSA	*Arabidopsis thaliana*	eudicots, rosids, Brassicales
AtPKSB	*Arabidopsis thaliana*	eudicots, rosids, Brassicales
Os10g0484800 (YY2)	*Oryza sativa*	monocots, Poales
Os07g0411300	*Oryza sativa*	monocots, Poales
** Embryophyte type III PKS (non-ORS, non-ASCL)**		
**Takakia Tle2c05338.1**	***Takakia lepidozioides***	**Bryophyta, Takakiales**
PpCHS	*Physcomitrium patens*	Bryophyta, Funariales
Mp4g23190	*Marchantia polymorpha*	Marchantiophyta, Marchantiales
AANG010604	*Anthoceros angustus*	Anthocerotophyta, Anthocerotales
MsCHS2	*Medicago sativa*	eudicots, rosids, Fabales
AhSTS	*Arachis hypogaea*	eudicots, rosids, Fabales
Gh2PS	*Gerbera hybrid cultivar*	eudicots, asterids, Asterales

## Extended Data

Description: Fig. S1 Amino acid sequences of plant type III polyketide synthases included in the phylogenetic tree ( Figure 1 ). Resource Type: Dataset. DOI: 10.22002/1tdp3-92698
